# Infant Feeding, Vitamin D and IgE Sensitization to Food Allergens at 6 Years in a Longitudinal Icelandic Cohort

**DOI:** 10.3390/nu11071690

**Published:** 2019-07-23

**Authors:** Birna Thorisdottir, Ingibjorg Gunnarsdottir, Anna Gudrun Vidarsdottir, Sigurveig Sigurdardottir, Bryndis Eva Birgisdottir, Inga Thorsdottir

**Affiliations:** 1 Unit for Nutrition Research, Faculty of Food Science and Nutrition, School of Health Sciences, University of Iceland and Landspitali University Hospital, 101 Reykjavik, Iceland; 2 Faculty of Sociology, Anthropology and Folkloristics, School of Social Sciences, University of Iceland, 101 Reykjavik, Iceland; 3 Department of Immunology, Landspitali University Hospital, 101 Reykjavik, Iceland; 4 Faculty of Medicine, University of Iceland, 101 Reykjavik, Iceland; 5 The Allergy Outpatient Department, Landspitali University Hospital, 108 Reykjavik, Iceland; 6 School of Health Sciences, University of Iceland, 101 Reykjavik, Iceland

**Keywords:** Nordic diet, infants, children, recommendations, IgE sensitization, breastfeeding, complementary feeding, solid food, vitamin D

## Abstract

Nordic Nutrition Recommendations (NNR) recommend exclusive breastfeeding until 6 months, partial breastfeeding until 1 year or longer and irrespective of breastfeeding, avoiding solid foods before 4 months. Strong evidence was found for benefits of breastfeeding regarding growth and infections but limited/inconclusive evidence regarding atopic disease and asthma. Vitamin D is of special interest in the Nordic diet. The aim of this prospective study was to compare infant feeding and vitamin D between immunoglobulin E (IgE) sensitized (*n* = 14) and non-sensitized (*n* = 130) children at 6 years. Information on diet and vitamin D supplement use were collected with dietary recall (<5 months), 1-d food records (5 and 6 months) and 3-d weighed food records (12 months and 6 years). Serum-specific IgE-antibodies against milk, egg, cod, wheat, soy and peanut (cut-off specific IgE ≥ 0.35 kUA/L) were measured at 6 years and serum 25-hydroxyvitamin D at 12 months and 6 years. At 4 months, 57% of IgE sensitized vs. 23% of non-sensitized children (*p* < 0.01) had received solid food. At 12 months, IgE sensitized children had a lower intake of vitamin D (median (25th, 75th percentiles): 3.9 μg/d (3.2, 7.2) vs. 8.1 μg/d (4.4, 12.3), *p* = 0.03) and at 6 years, fewer used vitamin D supplements regularly (23% vs. 56%, *p* = 0.03). Introduction of solid foods prior to 4 months increased the odds of IgE-sensitization, *OR* = 4.9 (95%, CI = 1.4–16.6) and vitamin D supplement at 6 years decreased the odds of IgE-sensitization, *OR* = 0.2 (95%, CI = 0.1–0.98), adjusting for maternal smoking. These observations support the NNR in their recommendation against introducing complementary solid foods before the age of 4 months. Furthermore, they support encouraging vitamin D intake for young children at northern latitudes.

## 1. Introduction

Dietary approaches to preventing food allergies are currently the topic of much research and may have implications for infant nutrition recommendations [1,2]. The Nordic Nutrition Recommendations (NNR) recommend exclusive breastfeeding until the infant is about 6 months old [3]. The frequency of breastfeeding is high in the Nordic countries: Data from 2006–2012 show that about 95% of newborns are breastfed and 68%–85% still receive any amount of breastmilk at the age of four months [4]. The most recent data from Denmark, Norway and Sweden, show similar values for breastfeeding [5,6,7]. Most Nordic infants, however, receive complementary foods before the age of 6 months in Denmark, Finland, Iceland, Norway and Sweden. At the age of 4 months, 23%–63% are exclusively breastfed, falling to 0%–12% at the age of 6 months [4]. The Nordic Nutrition Recommendations on breastfeeding state that some infants will need complementary feeding before 6 months of age, but they emphasize that solid food should not be introduced before the age of 4 months [3]. While most official bodies recommend exclusive breastfeeding for either the first 6 months or at least 4–6 months for health-related endpoints [3,8,9,10], there is a debate regarding the optimal age of introduction of solid food as primary prevention to reduce atopy and allergy [1]. The systematic literature review on breastfeeding prepared for the Nordic Nutrition Recommendations gave the lowest grade of evidence for any preventive effects of breastfeeding on the risk of atopic diseases in children, so no conclusions were drawn, while for most other undesirable outcomes there was convincing or probable evidence for the protective effects of breastfeeding [4]. Similarly, recent systematic literature reviews found the data inconclusive regarding the effect of breastfeeding on the risk of atopic diseases [11,12].

Due to the northern latitude, vitamin D is of special importance in the Nordic countries. In the NNR as well as national guidelines in all Nordic countries, parents are advised to give vitamin D supplements (10 μg/d) to their infants [3]. The importance of vitamin D supplement intake for vitamin D status at 12 months and 6 years of age in an Icelandic longitudinal cohort has been reported [13,14]. While vitamin D deficiency in infancy or childhood has been associated with higher immunoglobulin E (IgE) levels and sensitization to food allergens [15,16], other studies suggest that infant vitamin D supplementation increases the risk of later atopy and allergy [17,18,19].

The detection of serum-specific IgE to common food allergens indicates a state of sensitization, although in isolation it does not prove the existence of clinical allergy [20]. Studies have suggested that about half of sensitized children have a food allergy, clinically proven by food challenge [21]. However, according to a model by Grimshaw et al., primary prevention of allergies should focus on preventing immunological sensitization [2]. Therefore, we decided to test for serum-specific IgE to common food allergens at 6 years in the Icelandic longitudinal cohort with detailed information of the timing of solid food introduction and repeated information on the use of vitamin D supplements throughout the first year of life and in a follow-up at 6 years. Our aim was to compare feeding in infancy and vitamin D intake between sensitized and non-sensitized children.

## 2. Materials and Methods 

### 2.1. Participants and Design

Previous publications have described in detail the study population (a random sample of 250 Icelandic infants born in 2005), recruitment and data collection throughout the first year of life and at follow-up at 6 years [22,23]. The inclusion criteria were Icelandic (Caucasian) parents, singleton birth, 37–41 weeks gestational length, birth weight within 10th–90th percentiles, absence of birth defects or congenital long-term diseases and regular antenatal care of the mother. The current study includes 144 children who participated in blood sampling in the follow-up at 6 years and had allergen-specific IgE levels analyzed (84% of the children in the follow-up). As previously described, children participating in the blood sampling did not differ from the original sample in birth size and breastfeeding [24] and the basic characteristics of subjects included in this study did not differ from other subjects in the follow-up. Informed written consent from the parents was obtained for the study and all participant information was processed with strict confidentiality. The study was conducted according to the Declaration of Helsinki, approved by the National Bioethics Committee (VSNb2005040019/037; VSNb2011010008/037) and registered at the Data Protection Authority (S2449/2005; S5099/2011) in Iceland. 

### 2.2. Dietary Assessments

Detailed information about infant feeding from birth until 4 months (17 weeks) including breastfeeding, the introduction of infant formula and solid food and use of vitamin D or other dietary supplements were gathered with one structured dietary recall facilitated by a trained interviewer (mean infant age at dietary recall 21 weeks). The exact age when the first infant formula and first solid food was introduced to the diet, along with the type of formula or solid was registered. At 5 and 6 months, non-weighed intake was registered for 1 day (24 h) and at 12 months and 6 years weighed food records were kept for 3 consecutive days (72 h), thereof two weekdays and one weekend day (either Sunday–Tuesday or Thursday–Saturday). All food and fluids were weighed on accurate scales (Philips type HR 2385, Hungary and Austria) with 1 g precision. Dietary supplements and medicines were also registered. An average daily consumption of food and nutrients was calculated using the Icelandic food composition database [25] which included infant products, such as porridges, purées and cereals. Total vitamin D intake included vitamin D from food and supplements. Vitamin D supplement doses were most often between 6 and 10 μg/d. Since the doses did not differ greatly in amount, participants were classified as using vitamin D supplements if they received any amount of cod liver oil or another type of vitamin D containing supplement.

### 2.3. Blood Sampling and Biochemical Analysis 

At 12 months and 6 years, venous blood samples were collected in the morning in a fasting state at Landspitali University Hospital by a certified pediatrician and centrifuged within 6 h of collection. Separated serum samples were stored at −80 °C at the biobank at Landspitali until analyses were carried out by biomedical scientists at Landspitali for serum 25(OH)D at 12 months (April 2013), serum 25(OH)D at 6 years (December 2013) and serum-specific IgE to food allergens at 6 years (January 2014). 

Serum 25(OH)D levels were analyzed using an electro-chemiluminescence immunoassay (Elecsys Vitamin D total assay, Modular Analytics E170, Roche Diagnostics, Mannheim, Germany) (measuring range 7.5–175.0 nmol/L, precision 0.1 nmol/L). Tests were performed according to the manufacturer’s instructions [26]. 

Specific IgE levels were analyzed using ImmunoCAP fluoroenzyme immunoassay (Phadia 250, Thermo Scientific, Uppsala, Sweden) (precision 0.01 kUA/L) for ImmunoCAP^®^ allergen Fx5, a food mix test detecting specific IgE to six major food allergens in children (cow’s milk, egg white, cod, wheat, soybean and peanut). Tests were performed according to the manufacturer’s instructions and classified as a positive test, using the term ‘IgE sensitized’, when specific IgE ≥ 0.35 kUA/L. In the case of a positive test, specific IgE was further analyzed for each of the 6 major food allergens.

### 2.4. Other Variables

Background characteristics were chosen based on their availability in the original data and possible associations with sensitization according to literature, e.g., season of birth [27], urban living [28], firstborn child [29] and parental smoking during infancy [30,31]. Prior analyses of the data have shown maternal education and maternal BMI to be the variables most associated with breastfeeding duration and adherence to infant diet recommendations [32]. 

Information on parental variables including age, education, self-reported weight and height (from which BMI was calculated) and smoking after birth, as well as information on the infant’s siblings, was gathered from structured questionnaires answered by parents (usually the mother) when the children were 12 months old. Parents’ education was defined as the highest level of completed education categorized as basic education (finished elementary school) or longer based on earlier publications [32]. Birth months were categorized as ‘winter/spring’ (November–April) and ‘summer/autumn’ (May–October) based on the expected contribution of sunlight exposure to maternal vitamin D status around birth [13]. During follow-up at 6 years of age, parents answered another questionnaire including questions on children’s regular use of antihistamines and asthma inhalers. The questionnaires were constructed for the primary outcomes of the study (dietary intake and growth) and did not include questions about family history of atopy or maternal vitamin D supplementation during pregnancy or lactation. 

Information about weight, length and head circumference was gathered from maternity wards (birth measurements) and primary healthcare facilities (as close to ages 2, 6 and 12 months as possible). Weight was registered with 5 g precision, length measured on length boards and registered with 0.1 cm precision and head circumference was registered with 0.1 cm precision. At follow-up, (mean age 73.4 ± 3.2 months) weight (Marel M series 110, Iceland) (precision 0.1 kg) and height (Ulmer stadiometer, Professor Heinze, Busse design, Ulm, Germany) (precision 0.1 cm) were measured at Landspitali University Hospital. Children were classified as being a normal weight or overweight/obese at 6 years according to the World Health Organization’s (WHO) growth reference data for 5–19 years [33] and the age-specific International Obesity Task Force (IOTF) cut-off points used in previous publications from this cohort [34]. Gains in infant weight, length or head circumference were calculated for each participant as the difference in measurements between two time points.

### 2.5. Statistical Analyses

Statistical analyses were performed with SAS (Enterprise Guide 4.3; SAS Institute Inc, Cary, NC, USA). Variables were examined for normality using quantile-quantile plots. Categorical variables were presented as *n* (%) and a chi-square used for comparison between groups. Normally distributed continuous variables were presented as mean and standard deviations (SD) and compared using two-sided t-tests. Non-normally distributed continuous variables were presented as medians and 25th to 75th percentiles and compared using the non-parametric Mann–Whitney U-test. Logistic regression analyses were used to examine possible associations with sensitization at 6 years, adjusting for maternal smoking during infancy, and presented as the odds ratio (OR) with its 95% confidence intervals (CI). The level of significance was set at ≤0.05. 

## 3. Results

Fourteen 6-year-old children (10%) were IgE sensitized, thereof eight to cow’s milk only; two to cow’s milk and eggs; two to peanuts only; one to peanuts and eggs; and one to peanuts, wheat and soy. Four IgE sensitized children and one non-sensitized child were reported as regular users of asthma inhalers or antihistamine at 6 years. Table 1 presents the characteristics of the participants included in this study. Two IgE sensitized children (15%) vs. four non-sensitized children (3%) had mothers who reported smoking in the first year of the child’s life (*p* ≤ 0.05). Other background characteristics did not differ significantly between the groups. Looking at infant growth, IgE sensitized children gained significantly more weight and increased their head circumference significantly more in the first 2 months of life than non-sensitized children. At 2–6 months and 6–12 months (data not shown), no significant differences in weight gain or head circumference growth were observed. Depending on the applied method, 16% of non-sensitized vs. 36% of IgE sensitized (WHO method, non-significant (NS), *p* = 0.07) or 10% of non-sensitized vs. 29% of IgE sensitized (IOTF method, *p* = 0.04) were classified as overweight/obese at 6 years. 

**Figure 1:**
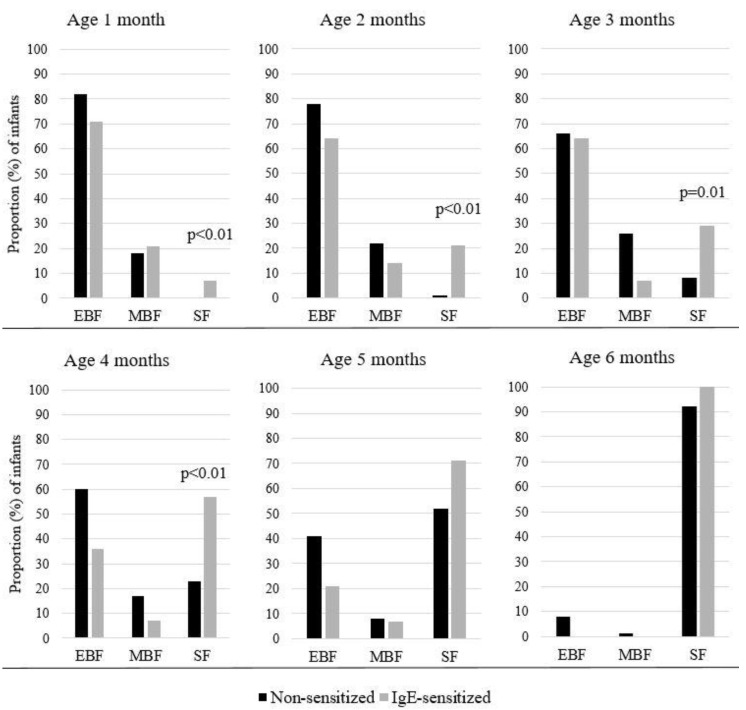
Proportion (%) of infants aged 1–6 months receiving exclusively breastmilk (EBF); mixed breastmilk and infant formula or exclusively infant formula (MBF); or any solid food (SF), shown for non-sensitized (*n* = 130) and IgE sensitized children (*n* = 14).

Breastfeeding initiation rate was high (99%) and 88% of infants were still breastfed when introduced to solid food. Neither exclusive breastfeeding, nor mixed breastmilk and infant formula or exclusively infant formula, in the first year differed according to sensitization status. The average duration of exclusive breastfeeding was 3.6 ± 1.9 months for non-sensitized children vs 2.9 ± 2.1 months for the IgE sensitized (NS). The average duration of any breastfeeding was similar for non-sensitized vs sensitized children. At 4 months 36% of IgE sensitized vs. 60% of non-sensitized children were exclusively breastfed (NS, *p* = 0.08). However, as shown in Figure 1, a greater proportion of IgE sensitized than non-sensitized children had been introduced to complementary solid food at or before 4 months of age (17 weeks) (57% vs. 23%, respectively, *p* < 0.01). Introduction of solid foods prior to 4 months increased the odds for sensitization, *OR* = 4.9 (95% CI = 1.4–16.6) when adjusted for maternal smoking. 

**Figure 2:**
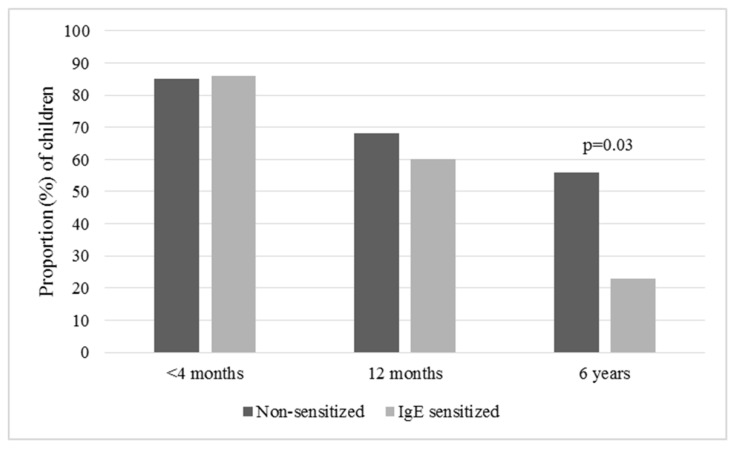
Proportion (%) of children of selected ages using vitamin D supplements, shown for non-sensitized (*n* = 130) and IgE sensitized (*n* = 14) children.

Total vitamin D intake at 12 months was lower among IgE sensitized than non-sensitized children, presented as median (25th, 75th percentiles): 3.9 μg/d (3.2, 7.2) vs. 8.1 μg/d (4.4, 12.3), respectively, *p* = 0.03. This could partly be explained by less but non-significant vitamin D supplement use among IgE sensitized children (see Figure 2) and partly by less consumption of vitamin D fortified formula (consumed by 30% of IgE sensitized vs. 68% of non-sensitized children at 12 months, *p* = 0.02). Higher vitamin D intake at 12 months was associated with a decreased odds for sensitization, *OR* = 0.8 (95%, CI = 0.7–0.996) when adjusted for maternal smoking. As shown in Figure 2, IgE sensitized children were less likely than non-sensitized children to use vitamin D supplements at 6 years of age (23% vs. 56%, *p* = 0.03). Vitamin D supplement use at 6 years was associated with a decreased odds for sensitization, *OR* = 0.2 (95%, CI = 0.1–0.98), when adjusted for maternal smoking. The median intake of vitamin D at 6 years was 3.1 μg/d (2.1, 3.6) vs. 5.3 μg/d (2.3, 12.4) among IgE sensitized vs. non-sensitized children, respectively (NS, *p* = 0.09). Mean serum 25(OH)D did not differ between IgE sensitized and non-sensitized children, at either 12 months (96.8 ± 33.6 vs. 99.3 ± 32.2 nmol/L, respectively) or 6 years (59.3 ± 15.9 vs. 56.0 ± 16.7 nmol/L, respectively). It should be noted that only half of participants had serum 25(OH)D measured at 12 months (69 non-sensitized and 7 IgE sensitized). 

## 4. Discussion

In this population-based study of Icelandic children, solid food introduction prior to 4 months was more common and total vitamin D intake at 12 months was lower among IgE sensitized vs. non-sensitized children. Furthermore, vitamin D supplement use at 6 years was less common among IgE sensitized children. Logistic models adjusted for maternal smoking (the only background variable that differed significantly according to sensitization), confirmed these results. 

The results add to the present literature about early life exposures on IgE outcomes in childhood, although this study is small and cannot determine causality. Also, a 9.7% IgE sensitization ratio at 6 years is higher than found in other studies of Icelandic children, measured at a younger age [35,36,37]. Although we did not have clinical information on food allergies, increased serum levels of specific IgE detected by immunoassay are commonly associated with a higher likelihood of clinical atopic disease or food allergy [38,39,40] and the prevention of sensitization is considered important for the prevention of food allergies [2]. Therefore, our results of 57% of IgE sensitized vs. 23% of non-sensitized children having been introduced to solid food at 4 months support the Nordic Nutrition Recommendations advice to delay complementary feeding until the infant is at least 4 months of age. They also seem to support the current vitamin D supplementation recommendations in Iceland and the Nordic countries. We did not observe significant differences in any or exclusive breastfeeding between IgE sensitized and non-sensitized children, although there was a trend (*p* = 0.08) suggesting that IgE sensitized children had lower rates of exclusive breastfeeding in infancy compared to non-sensitized children (36% vs. 60% at 4 months, respectively).

Literature suggests that by 4 months, the infant’s gut is sufficiently developed to receive and digest solid food or any food beyond breastmilk and infant formula [41,42]. Associations between the reduced relative abundance of potentially immunomodulatory gut bacteria and the development of IgE-associated eczema have also been suggested [43]. While some studies [44,45,46,47] but not all [48,49] propose that introduction of allergenic solid food from 3–4 months of age is safe and may lead to tolerance and protection against IgE-mediated food allergy, associations between solid food introduction prior to 4 months, faster infant growth and childhood obesity have been reported [50,51,52,53,54]. Our findings indicate interesting differences between the IgE sensitized and non-sensitized children in growth rate in infancy and perhaps also in the prevalence of body weight above the normal reference value at 6 years. Infant feeding (breastfeeding or early introduction of solid foods) may be a confounder in this, e.g., through higher infant energy and/or protein intake. Faster weight gain in infancy has been associated with an increased risk of allergic rhinitis [55], asthma [56,57,58,59], impaired lung function [60,61,62] and wheezing [55,63]. To the best of our knowledge, other studies have not observed the trend we see on less gain in postnatal head circumference among IgE sensitized children compared to their non-sensitized peers. It has been hypothesized that childhood obesity may promote immunological changes increasing the risk for allergy [64] but the evidence remains uncertain [65]. Previous papers from the currently studied population-based Icelandic cohort born in the year 2005, with extensive and detailed information on both dietary intake and growth throughout the first year of life and follow-up at 6 years of age, have shown associations between infant nutrition and growth up to 6 years. Solid food prior to 6 months of age and high protein intake and especially animal protein intake at 9–12 months has been associated with a body mass index above the normal range at the age of 6 years [23,66,67]. 

A Cochrane review from 2016 found no evidence to disagree with recommendations for healthy infants of exclusive breastfeeding for the first 6 months [68]. A more recent systematic review found moderate evidence suggesting that a short breastfeeding duration, or no breastfeeding, was associated with a higher risk of childhood asthma [11]. The same review found limited evidence for the effect of either a short breastfeeding duration or no breastfeeding on food allergies, allergic rhinitis and atopic dermatitis [11]. Another systematic literature review found limited to strong evidence, depending on the food in question, to suggest that introducing allergenic foods after 4 months of age does not affect the risk of food allergy and atopic dermatitis/eczema but may decrease the risk of later peanut and egg allergies [12]. Findings summarized in these reviews, and other papers, have resulted in changes to the older dietary advice about avoiding allergenic foods during the first year of life. Further studies are needed with a focus on diet in infancy and atopic disease, but our results on solid food introduction harmonize with these recent systematic reviews.

Vitamin D intake is important for all infants as well as children and adults with little sun exposure in all Nordic countries. Official recommendations in Iceland put special emphasis on vitamin D intake from the diet and supplements due to the country’s geographical location at 63–66° N, resulting in limited endogenous vitamin D synthesis for many months of the year [69,70]. It has been hypothesized that the relationship between vitamin D status and allergy may not be linear and that both low and high vitamin D status may be associated with elevated IgE levels [71]. While childhood vitamin D deficiency has been associated with increased asthma risk [72] and higher levels of IgE sensitization [15,16], other studies, from Finland, Sweden and Australia suggest that infant vitamin D supplementation may increase the risk for atopy and allergy later in life [17,18,19]. In the Northern Finland Birth Cohort 1966, the majority of infants used vitamin D supplements in doses of 50 μg/d [17], which is considered the upper limit for children, and too high in the first year of life [3]. In our cohort, no 12-month-old infant was considered vitamin D deficient (25-hydroxyvitamin D < 30 nmol/L) and vitamin D intake from diet and supplements combined did not exceed 25 µg/d in infancy or at 6 years [13,14]. A recent Icelandic study reported associations between postnatal cod liver oil consumption and decreased food sensitization and clinical food allergy in infants [35]. The present study found lower total vitamin D intake at 12 months and lower vitamin D supplement use (mainly cod liver oil) at 6 years among IgE sensitized children compared to non-sensitized. Previous work on this data has shown correlations between vitamin D supplement use in late infancy and at 6 years [73]. It is possible that children using vitamin D supplements at 6 years have been more likely than others to use vitamin D supplements from 1–6 years of age and it may therefore be viewed as a proxy for vitamin D supplement use in early childhood. We did not see a difference in vitamin D status by sensitization, but the small sample size may have weighted very heavily there, especially if the relationship between vitamin D and sensitization may not be linear [71]. We did not have information about maternal vitamin D intake during pregnancy or lactation. 

Cod liver oil is a good source of both vitamin D and n-3 PUFA. Vitamin D modulates the action of immune cells, such as T and B cells, monocytes and dendritic cells, in an interplay between the innate and adaptive immune systems [74]. We cannot exclude an effect from the intake of n-3 PUFA on IgE sensitization in the current study [75].

The strength of our study lies in the detailed longitudinal data on infant feeding, vitamin D supplement use and growth from birth to 6 years in a population-based sample, which makes it an important contribution to the literature. The thorough and direct methods of data collection decrease unexplained variabilities and increase the validity of the data. For the investigated population at northern latitudes the results on vitamin D intake are important. 

We acknowledge that our study is limited by the number of participants, resulting, for example, in wide confidence intervals, and results should therefore be cautiously interpreted, but the cohort’s size was sufficiently large for the findings we observe. There is no reason to think the sample selection process influenced the results or affected feeding practices or vitamin D supplementation. Also, the representativeness of the sample is good. Therefore the significant effects or associations observed do firmly exist, although other associations cannot be excluded. The inclusion criteria; born at term, Icelandic (Caucasian) parents and absence of disease, must however be kept in mind when interpreting the results and may limit the generalizability of the findings.

## 5. Conclusions

The results support the NNR on not introducing complementary solid food before the age of 4 months and encouraging vitamin D intake from diet and supplements for Nordic infants and children. The results also indicated interesting differences between the IgE sensitized and non-sensitized children in growth rate in infancy and perhaps also in prevalence of body weight above the normal reference value at 6 years, which warrant further studies. Although not significant, we observed lower rates of exclusive breastfeeding in infancy for IgE sensitized children compared with non-sensitized children, which could be investigated in a larger sample. 

## Figures and Tables

**Table 1:** Characteristics of the participants included in the study (*n* = 144).

	Non-Sensitized *n* = 130	IgE Sensitized *n* = 14	*p*
**Infant information**			
Boys, *n* (%)	65 (50)	9 (64)	0.31
Birth in winter/spring, *n* (%)	70 (54)	8 (57)	0.81
Urban living, *n* (%)	91 (70)	11 (79)	0.50
First born, *n* (%)	42 (36)	2 (17)	0.18
Exclusive breastfeeding, months	4 (2, 5)	4 (0, 4)	0.18
Any breastfeeding, months	8 (6, 10)	8 (7, 11)	0.97
Weight gain 0–2 months, kg	1.8 ± 0.5	2.2 ± 0.4	0.04
Weight gain 2–6 months, kg	2.4 ± 0.7	2.3 ± 0.7	0.59
Length gain 0–6 months ^1^, cm	16.8 ± 2.0	17.8 ± 1.7	0.09
Head circumference gain 0–2 months, cm	4.2 ± 1.0	4.9 ± 1.2	0.02
Head circumference gain 2–6 months, cm	4.2 ± 0.7	3.9 ± 0.8	0.15
**Parent information ^2^**			
Maternal smoking, *n* (%)	4 (3)	2 (15)	0.05
Paternal smoking, *n* (%)	18 (16)	3 (23)	0.49
Maternal age, years	31.3 ± 4.8	33.2 ± 5.4	0.18
Paternal age, years	34.1 ± 5.7	36.5 ± 6.6	0.16
Maternal BMI, kg/m^2^	23.5 (21.4, 26.5)	26.6 (21.8, 33.2)	0.21
Paternal BMI, kg/m^2^	26.0 (24.3, 28.1)	26.6 (23.4, 28.7)	0.82
Basic education mother ^3^, *n* (%)	19 (16)	4 (31)	0.19
Basic education father ^3^, *n* (%)	26 (23)	4 (31)	0.52

Data presented as *n* (%), mean ± SD or median (25th, 75th percentiles). Chi-square, two-sided *t*-test or Mann–Whitney U-test used for comparison between groups. ^1^ Length gain 0–2 months and 2–6 months not reported due to a large number of missing values for length at 2 months (missing *n* = 56). ^2^ From questionnaire answered when the infant is 12 months old. ^3^ Elementary school (10 years in school) is the highest level of completed education.
